# Chronic inflammatory demyelinating polyneuropathy. A case description

**DOI:** 10.1002/ccr3.9217

**Published:** 2024-08-05

**Authors:** Garcia‐Castillo María Ariana

**Affiliations:** ^1^ Hospital Carlos Andrade Marin Quito Ecuador

**Keywords:** central nervous system involvement, chronic inflammatory demyelinating polyneuropathy, intravenous immunoglobulins, visual evoked potentials

## Abstract

Patients affected by chronic inflammatory demyelinating polyradiculoneuropathy require close follow up due to the neuronal demyelination along with axonal degeneration associated with the disease process, giving the opportunity to the medical team of adequating therapeutics and other medical interventions, according to the evolution of the symptoms, to prevent irreversible axonal degeneration.

## Graphical abstract

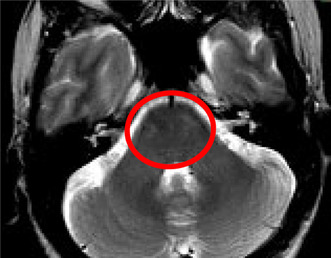

## 1 INTRODUCTION

Chronic inflammatory demyelinating polyneuropathy (CIDP) is clinically defined as a “chronically progressive, stepwise or recurrent proximal and distal weakness and sensory dysfunction of all extremities, developing over at least 2 months, with absent or reduced tendon reflexes in all limbs and sometimes with cranial nerve involvement.”^1^

CIDP is an acquired autoimmune disorder directed against the myelin sheath of peripheral nerves.^1^ It was initially characterized as chronic inflammatory polyradiculoneuropathy by Dyck et al. in 1975, but cases consistent with probable CIDP were described as early as 1958.^2^, ^3^

CIDP is difficult to diagnose, but early diagnosis can be crucial to prevent permanent nerve damage.^4^

Although CIDP is the most common treatable chronic neuropathy worldwide, it is still a rare disease.^5^

The reported prevalence of CIDP ranges from 0.7 to 10.3 cases per 100,000 people.^6^ There is a male predominance, with a gender rate ratio ranging from 1.5 to 4. CIDP primarily affects adults and the incidence rises with advancing age. The median age of onset is not well established. No specific predisposing risk factors for CIDP have been clearly identified.^6^, ^7^

There are several clinical presentations, and sensory dysfunction is frequently present, most usually affecting joint position, and vibration submodalities. Wasting is not prominent early in the disease. There are atypical forms, such as multifocal acquired demyelinating sensory and motor neuropathy (MADSAM, or Lewis– Sumner syndrome), pure sensory or pure motor CIDP, and focal or distal forms (distal acquired demyelinating sensory polyneuropathy (DADS)).^8^

Most people with CIDP have a progressive rather than a spontaneously relapsing and remitting course, with a variable balance between motor and sensory symptoms. The American Academy of Neurology established diagnostic research criteria for CIDP in 1991.^9^

However, there is still no gold standard set of diagnostic criteria for the electrophysiologic identification of demyelination, or for the clinical diagnosis of CIDP and its variants, even though multiple sets of diagnostic criteria have been published.^10^

Differences between these sets are related to definitions of the clinical picture, the requirements for nerve biopsy, electrodiagnostic criteria for demyelination, and the number of features required to make the diagnosis. The plethora of criteria sets for CIDP illustrate the difficulty of developing precise standards for problems that have multiple variations.

When independently validated in a retrospective study, the 2006 EFNS/PNS criteria had a sensitivity and specificity of 81 and 97 percent, respectively.^11^ The most frequently used CIDP criteria in clinical practice and research are the revised European Federation of Neurological Societies/Peripheral Nerve Society 2021 criteria.^12^

These and other proposed criteria typically comprise a combination of clinical and electrophysiological features (there have been 15 formal sets of published electrophysiological criteria for the diagnosis of CIDP^13^). Cases are classified as definite, probable, or possible, depending upon the number of criteria fulfilled. In most cases, finding a raised CSF protein without CSF leucocytosis further supports the diagnosis. Clear evidence of macrophage‐associated demyelination and remyelination, with or without a T‐cell inflammatory endoneurial infiltrate in a sensory nerve biopsy, remains the gold standard supportive criterion. There have been several validations of these diagnostic criteria, though none is 100% sensitive or specific; for example, the EFNS/PNS criteria show a positive predictive value of 97% and negative predictive value of 92%, and different validation cohorts give widely varying sensitivities and specificities.^14^

## 2 CASE DESCRIPTION

A 47‐year‐old woman presented to acute care with ataxia, nystagmus, vertigo, and diffuse burning tingling sensations, and weakness of all limbs, of greater frequency and intensity in the lower limbs.

Symptoms progressed over the course of 3 months.

Initially, weakness was most obvious when going up and down the stairs, but 2 weeks before going to the hospital, the weakness aggravated, with difficulty in walking.

### 2.1 Past medical, drug and habitual history

The patient reports occasional consumption of alcohol (at social celebrations), in an amount no greater than 90 cc. She had never used illicit drugs. She walks at least twice a week, for about an hour. The patient had no significant medical history, apart from two cesarean sections.

### 2.2 Family personal history

Three maternal aunts: Hashimoto's thyroiditis, with subsequent development of carcinoma (follicular in two, medullary in one).

Sister: multiple sclerosis, Hashimoto's thyroiditis.

Sister: rheumatoid arthritis, under biological treatment.

Grandmother: Akinetic Parkinson's.

#### 2.2.1 Physical examination

Glasgow Coma Score examination:

- Eye‐opening: 4: opens eyes spontaneously.
- Verbal response: 5: oriented, converses normally.
- Motor activity: 6: obeys commands.

##### 2.2.1.1 Muscular strength

According to Medical Research Council's scale (MRC scale), the following were observed:

Upper left limb proximal: 3/5 trapezius: 4−/5 wrist: 5/5 finger flexors: 4−/5.

Left lower limb proximal: 4−/5 knee flexor: 3/5.

Distal: 3/5.

Proximal right upper limb: 3/5 trapezius: 4−/5 wrist: 5/5 finger flexors: 4−/5.

Right lower limb Proximal: 4−/5 knee flexor: 3/5 Distal: 3/5.

##### 2.2.1.2 Surface sensitivity

Hypoesthesia of the internal face of the arm, forearm and hand (cubital).

Hypoesthesia of the lateral face of the right leg (external popliteal).

Preserved temperature and pain perception. Bilateral indifferent skin plantar reflex.

Deep sensitivity: kinesthesia, palesthesia normal in the four extremities.

Complex sensitivity: preserved steroagnosia, normal tactile discrimination.

Osteotendinous reflexes: generalized areflexia.

Coordination: altered bilateral finger nose test. Heel–Knee test normal. Truncal ataxia accented with closed eyes, in seating and bipedestation. Walking: lateralization on both sides during point‐heel, Romberg +.

**FIGURE 1:**
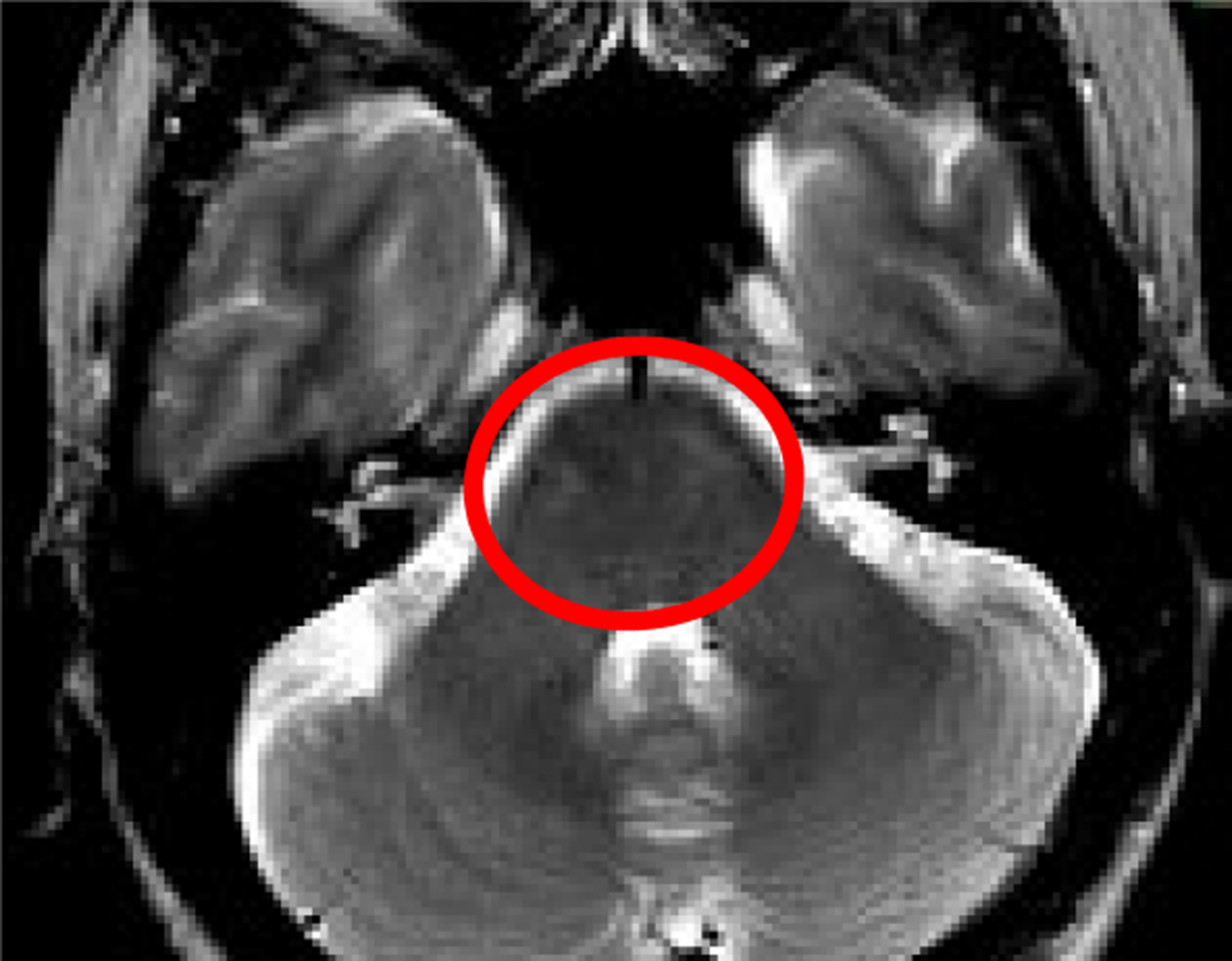
Scattered T2 hyperintensities in ventral pons (axial).

**FIGURE 2:**
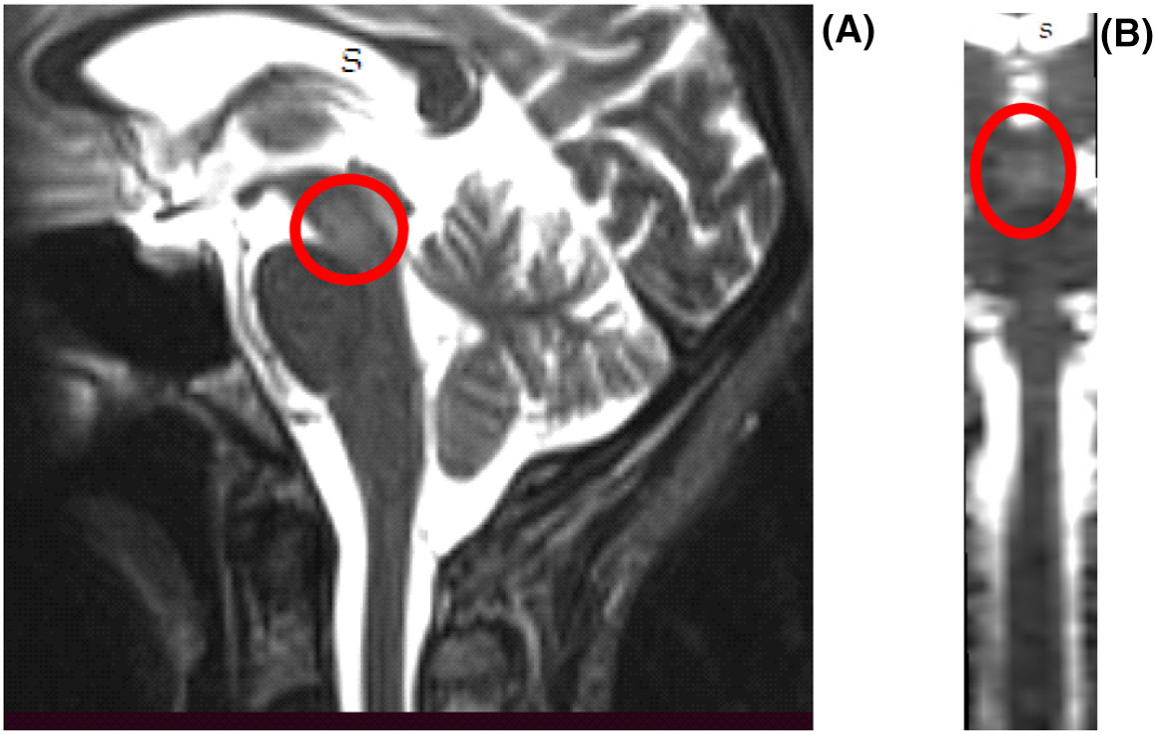
Scattered T2 hyperintensities in midbrain. (A) Sagittal. (B) Coronal reconstruction.

Following admission, MRI showed areas of scattered T2 and FLAIR signal hyperintensity in midbrain, anterior and posterior pons, in the distal medulla oblongata, and in the spinal cord, in C2‐C3 and C6, suspicious of demyelinating process (Figures 1 and 2).

Thickening in the distal course of the left C3 branch was observed.

In cerebrospinal fluid, glucose 54 mg/dL, protein 284 mg/dL, no red blood cells, and no white blood cells were observed. Gram staining was negative, and oligoclonal bands were absent.

Meningitis panel, including *Escherichia coli* K1, *Haemophilus influenzae*, *Listeria monocytogenes*, *Neisseria meningitidis*, *Streptococcus agalactiae*, *Streptococcus pneumoniae*, Cytomegalovirus, Enterovirus, Herpes simplex: virus 1 (HSV‐1), Herpes simplex virus 2 (HSV‐1), Herpes simplex virus 2 (HSV‐1), Human herpes 6 (HHV‐6), Human Parechovirus, Varicella Zoster Virus, *Cryptococcus neoformans*/*gattii*, was negative.

The serologic evaluation for immunological markers of disease, including ANA, anti‐DNA, anti‐ANCA PR3, anti‐ANCA MPO, C3, C4, anti‐RO, anti‐LA, B2 glycoprotein, anti‐TPO, antithyroglobulin was normal.

Rheumatoid factor was borderline.

Patient tested negative for endocrinological and tumoral markers of disease.

Markers for neurotropic viruses and *Toxoplasma* were negative.

Electromyography (EMG) showed axonal demyelinating polyradiculoneuropathy, abnormal distal latency with very low amplitude, disappearance of F waves, and numerous spontaneous potential (Table 1).

**TABLE 1:** Nerve conduction velocities in median, ulnar, and peroneal nerves.

Nerve	Left	Right
	velocity (m/s)	velocity (m/s)
Median	43.8	39.8
Ulnar	29.7	34.5
Peroneal	38.3	28.5

Motor nerve conduction velocities were reduced bilaterally, in nerves median, ulnar and peroneal, all of them with values under 44 m/s.

Acute denervation was determined in the supinator muscles, first dorsal interosseous of the hand and left anterior tibialis. Polyphasic potentials were obtained in the left trapezius.

Increased insertional activity in all the muscles was observed.

The recruitment patterns of the trapezius, supinator, first dorsal interosseous of the hand, gastrocnemius, tibialis, and left peroneal muscles are obtained with a reduced recruitment pattern in the right medial rump.

Neuro conduction: The median and ulnar right and left nerves, latency, amplitude and velocity values were obtained in normal values. Its F waves were obtained in a low percentage, but with latency within normal values.

Peroneal nerve: very low amplitude (0.89 mV in the left and 0.79 mV in the right). Latency and speeds in normal values. Its F waves were not possible to obtain.

Sural nerve: The left limb showed no potential.

Superficial peroneal nerve: The right limb was unresponsive.

As for this patient, she showed progressive weakness and numbness, both of which indicate alterations in the peripheral nervous system (PNS), then the EMG confirmed the damage of the PNS.

Subsequent studies revealed further deterioration of nerve conduction velocities especially in the legs, with acute denervation in muscles innervated by right C3‐C4 roots, bilateral C5‐C6 and right L4‐L5‐S1, with severe degree of involvement and neuro conduction study showing multiple motor mononeuropathy with axonal involvement of the bilateral suprascapular, bilateral radial, right ulnar and fibula or bilateral nerves, with a moderate degree of involvement.

Because of the sensory motor deficits, and cytological protein dissociation in cerebrospinal fluid, the patient was diagnosed with chronic inflammatory demyelinating polyneuropathy. It is to note that this patient had evidence of involvement of the central nervous system (CNS), given the MRI hyperintense areas, nystagmus, and cerebellar signs.

A 3‐day course of 1000 mg intravenous methylprednisolone was administered followed by oral corticosteroid treatment, prednisone 60 mg daily, diminishing 20 mg every 2 weeks to reach 20 mg, and then continuation to 10 mg, associating at this point with azathioprine 100 mg daily.

The patient also started a program of physiotherapy to improve muscle strength, function, and mobility, and minimize the shrinkage of muscles and tendons and distortions of the joints.

The numbness and weakness improved, and the patient could walk with the help of others. In a 6‐month follow‐up, the results showed there was still slight numbness of all limbs, and she could walk slowly, most of the time without help.

## 3 DIFFERENTIAL DIAGNOSES CONSIDERED AND DISCARDED

### 3.1 Charcot–Marie–tooth disease (CMT)

CMT is a hereditary neuropathy that can cause demyelination. In this case, there is no family history that could raise suspicion, nor musculoskeletal disorders.

### 3.2 Diabetic neuropathy

The exclusion criterion was diabetes, a common cause of neuropathy, and it can sometimes involve demyelination. However, in this case, there are not altered laboratory findings suggestions of diabetes.

### 3.3 Toxic neuropathies

The exclusion criterion was exposure to certain toxins, such as heavy metals or certain medications, can result in demyelinating polyneuropathy. There are not antecedents of ambiental nor personal exposure to toxins.

### 3.4 Paraneoplastic neuropathy

The exclusion criterion was some cancers can cause a paraneoplastic neuropathy, which may involve demyelination. A thorough cancer screening, including imaging studies and tumor markers, was done to the patient, excluding an underlying malignancy.

### 3.5 Vasculitis neuropathy

The exclusion criterion was vasculitis affecting peripheral nerves can lead to demyelination. Laboratory tests, such as erythrocyte sedimentation rate (ESR) and C‐reactive protein (CRP), as well as imaging studies like angiography, did not suggest vasculitis processes.

### 3.6 Infections

The exclusion criterion was certain infections, such as HIV or chronic inflammatory infections, can lead to demyelinating polyneuropathy. Serological and virological testing were performed in the patient to rule out infectious causes.

## 4 DISCUSSION

CIDP is a rare, immune‐mediated disorder in which an aberrant immune response causes demyelination and axonal damage of the peripheral nerves.^15^, ^16^ The exact etiology of CIDP remains unknown.^17^, ^18^ Patients experience progressive weakness, impaired sensory function in the legs and arms, loss of deep tendon reflexes (areflexia), and fatigue.^15^, ^18^, ^19^

CIDP has a substantial physical impact, with patients reporting pain, fatigue, and impaired physical functioning.^20^, ^21^, ^22^, ^23^, ^24^, ^25^, ^26^ Kuitwaard et al. assessed pain and fatigue, using the Numeric Pain Rating Scale (NPRS) and Fatigue Severity Scale (FSS) scores (*n* = 76), reporting that 17% and 74% of patients with CIDP reported severe pain and severe fatigue, respectively.^20^

The impact of CIDP may also extend beyond the physical burden of the disease to depression.^20^, ^22^, ^27^

The physical and mental manifestations of CIDP impair patient well‐being and quality of life.^6^, ^8^, ^10^ This may reflect the impact that chronic disabling conditions have on mental health.^28^

The study by Broers et al. reported incidence and prevalence for CIDP of 0.33 per 100,000 person‐years and 2.81 per 100,000 persons, respectively, which places CIDP among the rarest neuropathies.^1^, ^4^ Misdiagnosis can occur and patients may be treated inappropriately as a result.^29^, ^30^, ^31^

The diagnosis of CIDP can be made when patients fulfill a set of clinical, electrodiagnostic, and laboratory criteria.^1^, ^13^

However, the diagnosis can be difficult, in part because of the extensive list of differential diagnoses that mimic CIDP.^32^

Poorly performed nerve conduction studies, misinterpretation of their findings, and non‐adherence to electrodiagnostic criteria commonly lead to misdiagnosis.^32^, ^33^, ^34^

An incorrect diagnosis can also occur in patients reporting subjective improvement after treatment, or when minor elevation of the CSF protein concentration (probably not exceeding 1 g/L) is considered clinically relevant by the treating neurologist.^35^, ^36^, ^37^, ^38^

As for this patient, she showed progressive weakness and numbness, both of which indicate alterations in the PNS, confirmed for the EMG of the lower limbs, at the time she had clinical signs of alterations of the central nervous system, and lesions in the MRI that suggested demyelination.

The explanation of how CIDP can combine with central lesions, is still unclear. On the point of molecular biology: the myelin consists of inner and outer lipids and in‐between myelin sheath.

Approximately 15%–30% of myelin proteins are found in the CNS and the PNS. At least two common proteins which consist of the myelin have been found, resulting in similar autoimmune disorder in peripheral and central nerves in theory.^39^

When the blood–brain and blood‐nerve barriers are damaged, the antibodies cross the damaged barrier, resulting in an immune reaction in peripheral, and central nerves. So, if there appears immune‐mediated inflammatory response, there is a certain molecular basis of the mutual lesion. But the specific mechanism remains unclear.^40^

## 5 CONCLUSIONS

CIDP is a disabling immune‐mediated polyradiculoneuropathy with a typical phenotype and atypical variants, that involves both demyelination and axonal degeneration, with the balance being determined by disease duration and severity.^7^

The task of correctly diagnosing CIDP is often not easy, as there are many differential diagnoses and possible mimics. However, an early and accurate diagnosis is important to initiate treatment and to prevent further nerve damage.^41^, ^42^, ^43^

On the other hand, the consequences of over diagnosis are not trivial. From a safety perspective, the risks assumed from immunotherapy exposure are obvious. The therapies used to treat CIDP are not benign, nor are they (in most cases) cheap.^44^

Since central system lesions are an uncommon presentation of CIDP, we believe that clinicians need sufficient training to recognize this disease and be able to treat patients with appropriate care in a timely manner. Furthermore, because of the neuronal demyelination and axonal degeneration linked to the disease process, patients with CIDP need to be closely monitored. This allows the medical team to adjust therapies and other medical interventions in accordance with how the patient's symptoms develop in order to prevent irreversible axonal degeneration.

## AUTHOR CONTRIBUTIONS

**Garcia‐Castillo María Ariana:** Conceptualization; data curation; formal analysis; investigation; methodology; project administration; resources; validation; visualization; writing – original draft; writing – review and editing.

## FUNDING INFORMATION

No sources of funding were declared for this study.

## CONFLICT OF INTEREST STATEMENT

None declared.

## CONSENT

Written informed consent was obtained from the patient to publish this report in accordance with the journal's patient consent policy.

## Data Availability

The data that support the findings of this study are available from the corresponding author, MAGC, upon reasonable request.
